# S3‐Leitlinie Diagnostik und Therapie der Alopecia areata – Teil 2: Therapie, psychosoziale und kosmetische Unterstützung

**DOI:** 10.1111/ddg.70130

**Published:** 2026-08-04

**Authors:** Ulrike Blume‐Peytavi, Andria Constantinou, Tsenka Tomova‐Simitchieva, Adrian Tanew, Michael Sticherling, Hermann Girschick, Annika Vogt, Uwe Schwichtenberg, Uwe Gieler, André Märtens, Kerstin Zienert, Claudia Stenders, Ricardo Niklas Werner, Doris Wilborn

**Affiliations:** ^1^ Klinik für Dermatologie Venerologie und Allergologie Charité – Universitätsmedizin Berlin Corporate Member of Freie Universität Berlin Humboldt‐Universität zu Berlin und Berlin Institute of Health Berlin Deutschland; ^2^ Privatordination Wien Österreich; ^3^ Hautklinik Universitätsklinikum Erlangen Erlangen Deutschland; ^4^ Klinik für Kinder‐ und Jugendmedizin Vivantes Netzwerk für Gesundheit GmbH Klinikum im Friedrichshain Berlin Deutschland; ^5^ Derma Nord Hautarztpraxis, Deutschland; ^6^ Psychodermatology Competence Centre Universitätsklinikum Gießen Vitos Psychosomatik Gießen Gießen Deutschland; ^7^ Friseur Berlin Deutschland; ^8^ Alopecia Areata Deutschland e.V., Deutschland; ^9^ Klinik für Dermatologie Venerologie und Allergologie Division of Evidence‐based Medicine (dEBM) Charité – Universitätsmedizin Berlin Corporate Member of Freie Universität Berlin Humboldt‐Universität zu Berlin and Berlin Institute of Health Berlin Deutschland

**Keywords:** Alopezia areata, kreisrunder Haarausfall, Lebensqualität, topische und systemische Therapie, alopecia areata, circular hair loss, Quality of life, topical and systemic treatment

## Abstract

In diesem zweiten Teil der S3‐Leitlinie zur Diagnostik und Therapie der Alopecia areata (AA) stellen wir die zentralen Inhalte und Empfehlungen zur topischen und systemischen Therapie, zur Lebensqualität und zu Unterstützungsangeboten vor. Der separat publizierte erste Teil der Leitlinie umfasst die Definition der AA und Inhalte zur Epidemiologie, Diagnostik, Komorbidität, Risiko‐ und prognostischen Faktoren.

Die Applikation von Kortikosteroiden ist die einzige Intervention mit hohem Empfehlungsgrad für topisch anzuwendende Wirkstoffe. Alle anderen topischen Wirkstoffe, die zur Therapie der AA eingesetzt werden, können angeboten werden, haben einen niedrigen Empfehlungsgrad, wobei die Empfehlungsgrade für die einzelnen topischen Wirkstoffe in den Altersgruppen Kinder, Jugendliche und Erwachsene variieren.

Von den Wirkstoffen zur systemischen Therapie der AA haben nur die systemischen Kortikosteroide und die Januskinase‐Inhibitoren einen hohen Empfehlungsgrad, allerdings mit der Einschränkung, dass sich die Empfehlungen in der Anwendung nur auf Erwachsene beziehen. Kindern und Jugendlichen können Kortikosteroide, Methotrexat oder Januskinase‐Inhibitoren* (*bei entsprechender Zulassung für die Altersgruppe) ab einer schweren Form angeboten werden. Alle anderen systemischen Wirkstoffe sollten Kindern und Jugendlichen nicht angeboten werden. Die Leitliniengruppe empfiehlt, dass alle Patientinnen und Patienten mit allen Formen der AA, unabhängig vom Alter, über die Möglichkeiten eines Haarersatzes und über kosmetische Angebote informiert werden sollen.

## EINLEITUNG

Die vorliegende S3‐Leitlinie der Alopecia areata (AA) besteht aus zwei getrennt publizierten Teilen: Teil zwei (diese Publikation) basiert auf deren Langversion vom 18.09.2025 und wurde gemäß dem Regelwerk der AWMF Version 2.1 (2023),^1^ von Januar 2023 bis Oktober 2025 entwickelt. Er enthält die Empfehlungen und Hintergrundtexte der Kapitel Therapie, Lebensqualität, psychosoziale Unterstützung und kosmetische Angebote und sollte gemeinsam mit dem 1. Teil der Leitlinie zu Epidemiologie und Diagnostik genutzt werden. Patientenzielgruppe der Leitlinie sind Patienten mit einer AA in allen Altersgruppen, die Empfehlungen wurden spezifisch für Kinder, Jugendliche und Erwachsene formuliert.

Das dieser Veröffentlichung zugrundliegende Projekt wurde mit Mitteln des Innovationsausschusses beim Gemeinsamen Bundesausschuss unter dem Förderkennzeichen 01VSF22016 (S3 LL AA) im Zeitraum vom 01. Januar 2023 bis zum 30. Juni 2025 gefördert. Die Erstellung der S3‐Leitlinie folgte dem im Regelwerk der AWMF, Version 2.0, 2021,^2^ und der Version 2.1, 2023,^1^ beschriebenen Procedere.

Die methodischen Schritte der Leitlinienentwicklung wurden ausführlich im Leitlinienreport beschrieben (siehe auch Online‐Supplement und Webseite der AWMF: https://register.awmf.org/de/leitlinien/detail/013‐104. Die Leitlinie hat eine Gültigkeit von 5 Jahren (bis zum 17.09.2030) und soll nach diesem Zeitraum bei Änderungen in zentralen Themen aktualisiert werden.

## THERAPIE

Zur Behandlung der AA stehen topische und systemische Therapien zur Verfügung. Daher werden die Behandlungsmöglichkeiten der AA im Folgenden getrennt nach topischen und systemischen Optionen beschrieben. Die Leitliniengruppe hat einen Flow‐Chart nach Altersstufen entwickelt, der die Empfehlungen der Diagnostik und Therapie grafisch zusammenfasst.

**ABBILDUNG 1:**
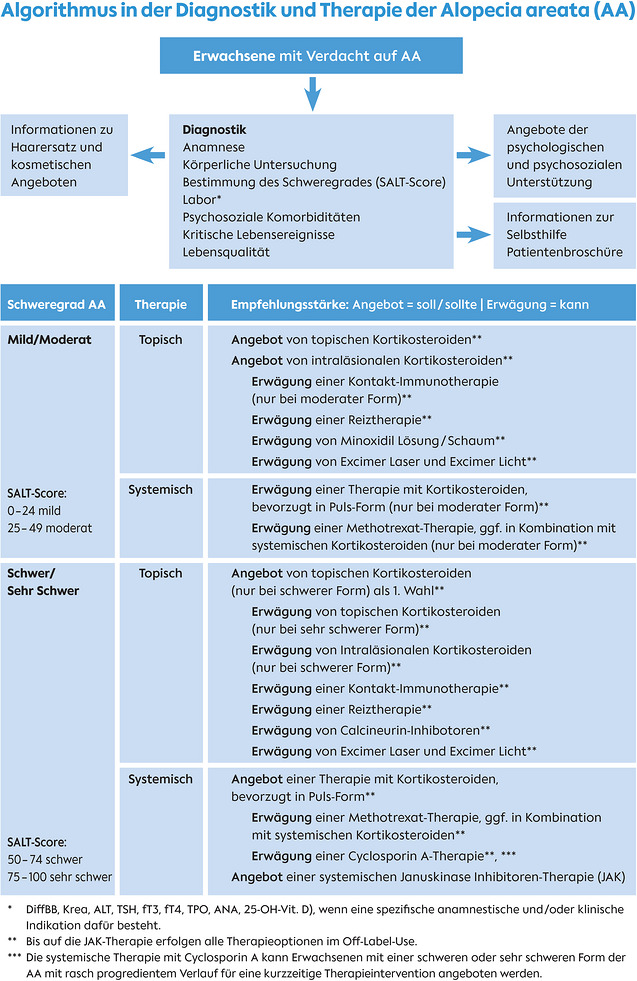
Algorithmus AA Kinder und Jugendliche.

Die Abbildungen 1 und 2 zeigen getrennt nach Altersstufen in Übersichtsform die Diagnostik und die Therapieoptionen jeweils separat für die Schweregrade mild/ moderat und schwer/sehr schwer der AA. Die Formulierung „Angebot“ spiegelt die Empfehlungsstärke *soll* oder *sollte* wider. Der Begriff „Erwägung“ weist auf eine „Kann‐Empfehlung“ hin.

**ABBILDUNG 2:**
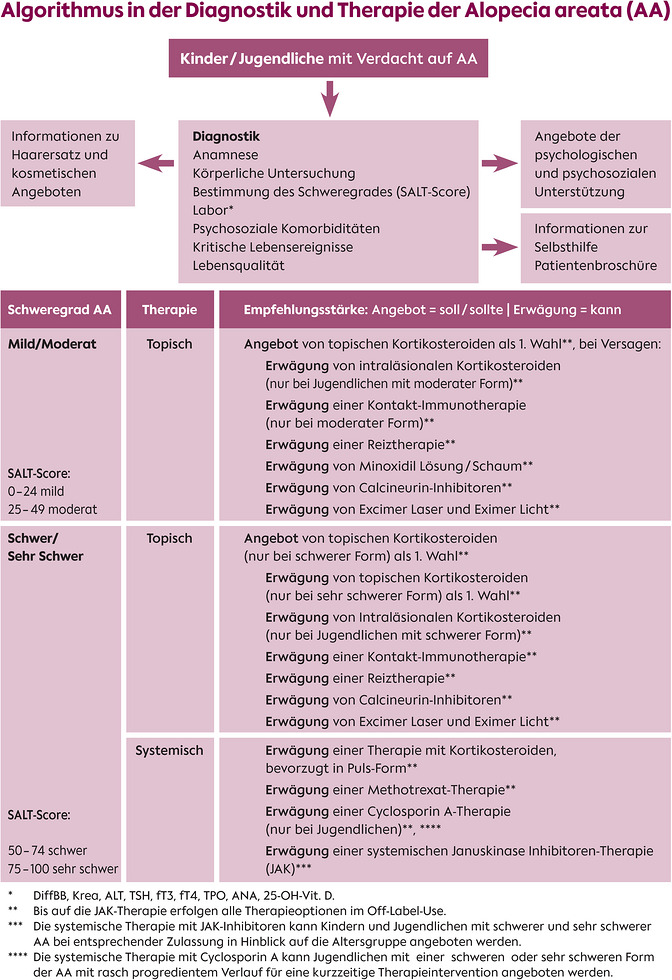
Algorithmus AA Erwachsene.

Tabelle 1 zeigt in einer Übersicht alle topischen Behandlungsmöglichkeiten, getrennt nach Altersgruppen und Schweregrad der AA mit den unterschiedlichen Empfehlungsstärken. Der Schweregrad der AA wird durch den SALT‐Score angegeben (*Severity of Alopecia Tool*).

**TABELLE 1:** Übersicht über alle Empfehlungen nach Wirkstoff – topische Therapie.

Wirkstoff	Schweregrad SALT‐Score	Kinder	Jugendliche	Erwachsene
Topische Applikation von Kortikosteroiden	Mild < 25 %	**↑↑**	**↑↑**	**↑↑**
	Moderat 25–49 %	**↑↑**	**↑↑**	**↑↑**
	Schwer > 50–75 %	**↑**	**↑↑**	**↑↑**
	Sehr schwer 76–100 %	**0**	**0**	**0**
	***Augenbrauen, Bart***	**↑**	**↑**	**↑**
Intraläsionale Applikation von Kortikosteroiden (ILC)	Mild < 25 %	**↓**	**↓**	**↑**
	Moderat 25–49 %	**↓**	**0**	**↑**
	Schwer > 50–75 %	**↓**	**0**	**0**
	Sehr schwer 76–100 %	**↓**	**↓**	**↓**
	***Augenbrauen, Bart***	Keine Empfehlung	**0** ^*^	**0** ^*^
Kontakt‐Immunotherapie (DCP/SADBE)	Mild < 25 %	**↓↓**	**↓↓**	**↓↓**
	Moderat 25–49 %	**0**	**0**	**0**
	Schwer > 50–75 %	**0**	**0**	**0**
	Sehr schwer 76–100 %	**0**	**0**	**0**
Reiztherapie mit Dithranol	Mild < 25 %	**0**	**0**	**0**
	Moderat 25–49 %	**0**	**0**	**0**
	Schwer > 50–75 %	**0**	**0**	**0**
	Sehr schwer 76–100 %	**0**	**0**	**0**
Minoxidil (Lösung oder Schaum)	Mild < 25 %	**0**	**0**	**0**
	Moderat 25–49 %	**0**	**0**	**0**
	Schwer > 50–75 %	Keine Empfehlung	Keine Empfehlung	Keine Empfehlung
	Sehr schwer 76–100 %	Keine Empfehlung	Keine Empfehlung	Keine Empfehlung
Calcineurininhibitoren	Mild < 25 %	**0**	**0**	Keine Empfehlung
	Moderat 25–49 %	**0**	**0**	Keine Empfehlung
	Schwer > 50–75 %	**0**	**0**	**0**
	Sehr schwer 76–100 %	**0**	**0**	**0**
	***Augenbrauen, Bart***	**↑**	**↑**	**↑**
Prostaglandin‐F2α‐Analoga	Mild < 25 %	**↓↓**	**↓↓**	Keine Empfehlung
	Moderat 25–49 %	**↓↓**	**↓↓**	Keine Empfehlung
	Schwer > 50–75 %	**↓↓**	**↓↓**	Keine Empfehlung
	Sehr schwer 76–100 %	**↓↓**	**↓↓**	Keine Empfehlung
	***Wimpern***	**0**	**0**	**0**
Thrombozytenreiches Plasma (PRP)	Mild < 25 %	**↓↓**	**↓↓**	Keine Empfehlung
	Moderat 25–49 %	**↓↓**	**↓↓**	Keine Empfehlung
	Schwer > 50–75 %	**↓↓**	**↓↓**	Keine Empfehlung
	Sehr schwer 76–100 %	**↓↓**	**↓↓**	Keine Empfehlung
JAK‐Inhibitoren	Mild < 25 %	**↓↓**	**↓↓**	**↓↓**
	Moderat 25–49 %	**↓↓**	**↓↓**	**↓↓**
	Schwer > 50–75 %	**↓↓**	**↓↓**	**↓↓**
	Sehr schwer 76–100 %	**↓↓**	**↓↓**	**↓↓**
Excimer‐Laser und Excimer‐Licht	Mild < 25 %	**0**	**0**	**0**
	Moderat 25–49 %	**0**	**0**	**0**
	Schwer > 50–75 %	**0**	**0**	**0**
	Sehr schwer 76–100 %	**0**	**0**	**0**
UVA1‐Phototherapie und Photochemotherapie (PUVA)	Mild < 25 %	**↓**	**↓**	**↓**
	Moderat 25–49 %	**↓**	**↓**	**↓**
	Schwer > 50–75 %	**↓**	**↓**	**↓**
	Sehr schwer 76–100 %	**↓**	**↓**	**↓**

* Angebot nur von in der Anwendung erfahrenen Ärztinnen und Ärzten.

### Watch‐and‐Wait

Das Vorgehen der Watch‐and‐Wait‐Option wird ausführlich im Kapitel 5.1 in der Langversion der Leitlinie beschrieben. Die Leitliniengruppe hat keine Empfehlung für dieses Vorgehen formuliert.

### Topische Therapie

#### Einführung

Die verschiedenen Wirkstoffe, die in den topischen Applikationsformen in der Therapie der AA zum Einsatz kommen, sind in der Regel zur Behandlung der AA in Deutschland nicht zugelassen, können jedoch im *Off‐Label‐Use* verwendet werden. Auf den *Off‐Label‐Use* wird in den Empfehlungen jeweils hingewiesen. Im Hintergrundtext der Langversion wird er aber nicht nochmals extra erwähnt. Laut Sozialgesetzbuch (SGB) Fünftes Buch (V) – Gesetzliche Krankenversicherung – (Artikel 1 des Gesetzes v. 20. Dezember 1988, BGBl. I S. 2477), § 34 Ausgeschlossene Arznei‐, Heil‐ und Hilfsmittel sind alle Medikamente, die „zur Verbesserung des Haarwuchses dienen“, von dieser Regelung betroffen, das heißt, dass keine Kostenübernahme durch die gesetzlichen Krankenkassen für diese Medikamente in der Therapie der AA erfolgt.^3^ Für eine Übernahme der Kosten bei Kindern, Jugendlichen und Erwachsenen kann versucht werden, individuelle Anträge auf Kostenerstattung bei den Krankenversicherungen zu stellen. Aufgrund der oben genannten Gesetzeslage wird die überwiegende Mehrheit der individuellen Anträge auf Kostenerstattung von den Krankenversicherungen jedoch abgelehnt.

Im Folgenden werden die Empfehlungen zu den einzelnen Wirkstoffen der topischen Therapie hier vorgestellt, jedoch nur ausgewählte Therapien werden ausführlich wie in der Langversion der Leitlinie beschrieben. Bei der ausführlichen Beschreibung konzentrieren wir uns dabei auf die neueren Therapien wie die Anwendung von Thrombozytenreichem Plasma (PRP), Licht‐ und Lasertherapie und die Anwendung von topischen Januskinase‐Inhibitoren.

### Allgemeine Therapiegrundsätze zu Kortikosteroiden

**Table:** 

	Allgemeine Therapiegrundsätze zu Kortikosteroiden	Empfehlungsstärke
**E1**	Zur topischen Therapie der AA im Bereich der Kopfhaut mit Kortikosteroiden *sollen* bei Jugendlichen und Erwachsenen bevorzugt Substanzen der Wirkstoffklassen III–IV (nach Niedner) mit einem hohen therapeutischen Index angewandt werden.	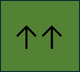
**E2**	Zur topischen Therapie der AA im Bereich der Kopfhaut mit Kortikosteroiden *sollen* bei Kindern bevorzugt Substanzen der Wirkstoffklassen II–III (nach Niedner) mit einem hohen therapeutischen Index angewandt werden.	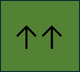
**E3**	Zur intraläsionalen Therapie der AA im Bereich der Kopfhaut mit Kortikosteroiden *sollen* 2,5–10 mg/ml Triamcinolonacetonid (0,1 ml/cm^2^, max. 100 cm^2^, streng intrakutan) angewandt werden.	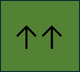
**E4**	Bei fehlendem Ansprechen *sollte* eine Behandlung mit topischen oder intraläsionalen Kortikosteroiden eine Behandlungsdauer von 3 Monaten nicht überschreiten.	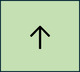
**E5**	Bei Therapieansprechen auf topische oder intraläsionale Kortikosteroide *sollte* diese Therapie individualisiert mit einer maximalen Anwendungsdauer von 12 Monaten fortgeführt werden.	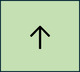
	Starker Konsens	**Konsensbasiert**

*Anmerkung*: die Empfehlungen zu topischen Kortikosteroiden beziehen sich auf die Applikation auf der Kopfhaut. Empfehlungen zu spezifischen Lokalisationen sind separat beschrieben.

### Topische Kortikosteroide

**Table:** 

	Empfehlung	Empfehlungsstärke
**E34**	Die topische Applikation von Kortikosteroiden *soll* Kindern mit einer milden oder moderaten Form der AA und Jugendlichen und Erwachsenen mit einer milden, moderaten oder schweren Form der AA im *Off‐Label‐Use* angeboten werden.	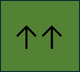
	Starker Konsens	**Evidenzbasiert**
	Referenzen: ^4^, ^5^, ^6^, ^7^, ^8^, ^9^, ^10^, ^11^, ^12^, ^13^, ^14^, ^15^	
	LoE: Level 2*, Level 2	
	Level 2*: von Level 1 auf Level 2 herabgestufte systematische Übersichtsarbeit, beziehungsweise Metaanalyse, wenn dort nur Beobachtungsstudien eingeschlossen waren (siehe Oxford 2011 LoE‐Schema^16^)	

**Table:** 

	Empfehlung	Empfehlungsstärke
**E35**	Die topische Applikation von Kortikosteroiden *sollte* Kindern mit einer schweren Form der AA im *Off‐Label‐Use* angeboten werden.	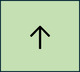
	Starker Konsens	**Evidenzbasiert**
	Referenzen: ^4^	
	*Level of evidence* (LoE): Level 2*	

**Table:** 

	Empfehlung	Empfehlungsstärke
**E36**	Die topische Applikation von Kortikosteroiden *kann* Kindern mit einer sehr schweren Form der AA mit Nagelbeteiligung und/oder Körperhaarverlust im *Off‐Label‐Use* angeboten werden.	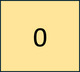
	Konsens	**Evidenzbasiert**
	Referenzen: ^4^	
	LoE: Level 2*	

**Table:** 

	Empfehlung	Empfehlungsstärke
**E37**	Die topische Applikation von Kortikosteroiden *kann* Jugendlichen und Erwachsenen mit einer sehr schweren Form der AA mit Nagelbeteiligung und/oder Körperhaarverlust im *Off‐Label‐Use* angeboten werden.	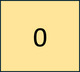
	Starker Konsens	**Konsensbasiert**

Die vollständigen Erläuterungen zur Wirksamkeit, Dosierung und besondere Hinweise sind in der Langversion der Leitlinie zu finden.

### Intraläsionale Kortikosteroide

*Anmerkung*: die Empfehlungen zu intraläsionalen Kortikosteroiden (ILC) beziehen sich auf die Applikation für die Kopfhaut. Empfehlungen zu spezifischen Lokalisationen sind extra beschrieben.

**Table:** 

	Empfehlung	Empfehlungsstärke
**E38**	Die intraläsionale Applikation von Kortikosteroiden *sollte* Kindern mit allen Formen der AA und Jugendlichen mit einer milden Form der AA *nicht* angeboten werden.	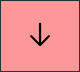
	Starker Konsens	**Evidenzbasiert**
	Referenzen: ^4^, ^17^	
	LoE: Level 2*, Level 4	

**Table:** 

	Empfehlung	Empfehlungsstärke
**E39**	Die intraläsionale Applikation von Kortikosteroiden *kann* Jugendlichen mit einer moderaten oder schweren Form der AA im *Off‐Label‐Use* angeboten werden.	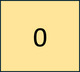
	Starker Konsens	**Evidenzbasiert**
	Referenzen: ^18^	
	LoE: Level 2	

**Table:** 

	Empfehlung	Empfehlungsstärke
**E40**	Die intraläsionale Applikation von Kortikosteroiden *sollte* Jugendlichen und Erwachsenen mit einer sehr schweren Form der AA *nicht* angeboten werden.	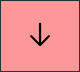
	Starker Konsens	**Konsensbasiert**

**Table:** 

	Empfehlung	Empfehlungsstärke
**E41**	Die intraläsionale Applikation von Kortikosteroiden *sollte* Erwachsenen mit einer milden oder moderaten Form der AA im *Off‐Label‐Use* angeboten werden.	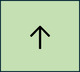
	Starker Konsens	**Evidenzbasiert**
	Referenzen: ^18^, ^19^, ^20^, ^21^, ^22^, ^23^, ^24^	
	LoE: Level 2	

**Table:** 

	Empfehlung	Empfehlungsstärke
**E42**	Die intraläsionale Applikation von Kortikosteroiden *kann* Erwachsenen mit einer schweren Form der AA im *Off‐Label‐Use* angeboten werden.	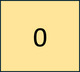
	Starker Konsens	**Konsensbasiert**

Die vollständigen Erläuterungen zur Wirksamkeit, Dosierung und besondere Hinweise sind in der Langversion der Leitlinie zu finden.

### Topische Immuntherapie

**Table:** 

	Empfehlung	Empfehlungsstärke
**E43**	Die Kontakt‐Immunotherapie (DCP/SADBE) *soll* Kindern, Jugendlichen und Erwachsenen mit einer milden Form der AA *nicht* angeboten werden.	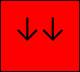
	Starker Konsens	**Konsensbasiert**

**Table:** 

	Empfehlung	Empfehlungsstärke
**E44**	Die Kontakt‐Immunotherapie (DCP/SADBE) *kann* bei Kindern mit einer moderaten, schweren oder sehr schweren chronischen Form der AA als individueller Heilversuch im *Off‐Label‐Use* erwogen werden.	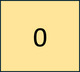
	Starker Konsens	**Evidenzbasiert**
	Referenzen: ^4^, ^25^, ^26^, ^27^, ^28^, ^29^, ^30^, ^31^, ^32^	
	LoE: Level 2*, Level 2	

**Table:** 

	Empfehlung	Empfehlungsstärke
**E45**	Die Kontakt‐Immunotherapie (DCP/SADBE) *kann* Jugendlichen und Erwachsenen mit einer moderaten, schweren oder sehr schweren Form der AA als individueller Heilversuch im *Off‐Label‐Use* angeboten werden.	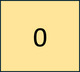
	Starker Konsens	**Evidenzbasiert**
	Referenzen: ^4^, ^25^, ^26^, ^27^, ^28^, ^29^, ^30^, ^31^, ^32^	
	LoE: Level 2*, Level 2	

Der Hersteller des Diphenylcyclopropenon (DCP)‐Wirkstoffs zur Anwendung am Menschen hat die Produktion in Deutschland eingestellt, sodass zurzeit das Präparat nicht zur Verfügung steht. Ob ein anderer Anbieter diesen Wirkstoff zur Anwendung am Menschen wieder herstellen wird, ist bisher nicht bekannt.

Die vollständigen Erläuterungen zur Wirksamkeit, Dosierung und besondere Hinweise sind in der Langversion der Leitlinie zu finden.

### Topische Reiztherapie

**Table:** 

	Empfehlung	Empfehlungsstärke
**E46**	Die Reiztherapie mit Dithranol *kann* Kindern und Jugendlichen mit allen Formen der AA im *Off‐Label‐Use* angeboten werden.	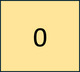
	Starker Konsens	**Evidenzbasiert**
	Referenzen: ^4^, ^25^	
	LoE: Level 2*	

**Table:** 

	Empfehlung	Empfehlungsstärke
**E47**	Die Reiztherapie mit Dithranol *kann* Erwachsenen mit allen Formen der AA im *Off‐Label‐Use* angeboten werden.	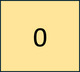
	Konsens	**Evidenzbasiert**
	Referenzen: ^32^, ^33^	
	LoE: Level 2	

Die vollständigen Erläuterungen zur Wirksamkeit, Dosierung und besondere Hinweise sind in der Langversion der Leitlinie zu finden.

### Topisches Minoxidil

**Table:** 

	Empfehlung	Empfehlungsstärke
**E48**	Die Anwendung von Minoxidil Lösung oder Schaum *kann* Kindern, Jugendlichen und Erwachsenen mit milder oder moderater AA zur Behandlung im *Off‐Label‐Use* angeboten werden.	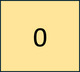
	Starker Konsens	**Evidenzbasiert**
	Referenzen: ^4^, ^29^, ^34^, ^35^, ^36^	
	LoE: Level 1, Level 2*, Level 2	

Die vollständigen Erläuterungen zur Wirksamkeit, Dosierung und besondere Hinweise sind in der Langversion der Leitlinie zu finden.

### Topische Calcineurininhibitoren

**Table:** 

	Empfehlung	Empfehlungsstärke
**E49**	Die topische Anwendung von Calcineurininhibitoren *kann* Kindern und Jugendlichen mit allen Formen der AA und Erwachsenen mit schwerer oder sehr schwerer AA im *Off‐Label‐Use* angeboten werden.	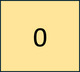
	Starker Konsens	**Evidenzbasiert**
	Referenzen: ^4^, ^7^, ^9^, ^10^, ^11^, ^12^, ^37^	
	LoE: Level 2*, Level 2	

Die vollständigen Erläuterungen zur Wirksamkeit, Dosierung und besondere Hinweise sind in der Langversion der Leitlinie zu finden.

### Topische Anwendung von Prostaglandin‐F2α‐Analoga

**Table:** 

	Empfehlung	Empfehlungsstärke
**E50**	Die topische Anwendung von Prostaglandin‐F2α‐Analoga *soll* Kindern, Jugendlichen und Erwachsenen mit allen Formen der AA zur Anwendung auf der Kopfhaut *nicht* angeboten werden.	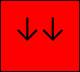
	Starker Konsens	**Evidenzbasiert**
	Referenzen: ^38^, ^39^	
	LoE: Level 2	

Die vollständigen Erläuterungen zur Wirksamkeit, Dosierung und besondere Hinweise sind in der Langversion der Leitlinie zu finden.

### Thrombozytenreiches Plasma (PRP)

**Table:** 

	Statement	
**S2**	Aufgrund der vorliegenden Studienlage *kann keine* Empfehlung für oder gegen die Anwendung von PRP bei AA ausgesprochen werden.	
	Starker Konsens	**Evidenzbasiert**
	Referenzen: ^31^, ^40^, ^41^, ^42^	
	LoE: Level 1, Level 2	

**Table:** 

	Empfehlung	Empfehlungsstärke
**E51**	Kindern und Jugendlichen *soll* die Anwendung von PRP zur Behandlung einer AA *nicht* angeboten werden.	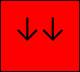
	Starker Konsens	**Evidenzbasiert**
	Referenzen: ^40^, ^41^	
	LoE: Level 1	

Die vollständigen Erläuterungen zu Wirksamkeit, Dosierung und besondere Hinweise sind in der Langversion der Leitlinie zu finden.

#### Auszug aus dem Abschnitt Wirksamkeit

Eine Metaanalyse mit vier eingeschlossenen randomisierten kontrollierten Studien (RCT; *randomized controlled trials*) und insgesamt 201 Patienten, die die intraläsionale PRP‐Therapie mit der intraläsionalen Triamcinolonacetonid (TA)‐Therapie vergleicht, zeigt, dass beim Vergleich im SALT‐Score nach 12 Wochen eine mittlere Differenz (MD) des SALT‐Scores von MD –2.04 (95 %‐Konfidenzintervall [KI] –4.72–0.65) für die TA erreicht werden konnte. Die Metaanalyse zeigt eine Überlegenheit der TA‐Behandlung im Vergleich zur PRP, das Ergebnis war jedoch statistisch nicht signifikant.^40^

In der systematischen Übersichtsarbeit von Tejapira et al. 2022 mit 23 Studien und 621 Patienten zeigt PRP bei leichten Fällen von AA eine Überlegenheit gegenüber Placebo in Bezug auf die Reduktion des SALT‐Scores, des Nachwachsens der Haare und des Rückgangs dystrophischer Haare. Bei schwereren AA‐Fällen (AT) erwies sich PRP als Monotherapie jedoch als unwirksam.^41^ Im Vergleich zu TA zeigen fünf Studien eine vergleichbare Wirksamkeit von PRP bei Patienten mit AA < 25 % oder einer Krankheitsdauer < 6 Monate. PRP war topischem 5%igem Minoxidil hinsichtlich der Verbesserung von dystrophischem Haar überlegen und hatte einen größeren Effekt auf den Haardurchmesser im Vergleich zur *Low‐Level*‐Laser‐Therapie (LLLT).^41^

In einer weiteren RCT, die nicht in der Metaanalyse von Meznerics et al. 2022 enthalten war, erwies sich die PRP‐Therapie ebenfalls als weniger wirksam im Vergleich zur intraläsionalen TA. In der TA‐Gruppe sprachen 90 % der Patienten auf die Behandlung an, während in der PRP‐Gruppe 73 % der Patienten ein Ansprechen zeigten (p < 0,05).^42^ Die Kombination von PRP mit DCP zeigte keine Überlegenheit gegenüber der DCP‐Monotherapie.^31^

Tejapira et al. 2022 diskutieren kritisch die folgenden Nachteile in den eingeschlossenen Studien ihrer Übersichtsarbeit: sie sehen in der kurzen Nachbeobachtungsdauer der Studien, dass die Sicherheit der PRP‐Therapie nicht angemessen beurteilt werden kann.^41^ Belege, die eine langfristige Wirksamkeit der Therapie zeigen, stehen noch aus ebenso wie geeignete Kriterien zur Auswahl der Patienten, die sich für die Therapie eignen. Hier sollten zukünftige Studien die aktuelle Heterogenität in der PRP‐Therapie in den Blick nehmen.

Trotz vielversprechender Ergebnisse in einigen Studien wird die klinische Anwendung von PRP durch den fehlenden Konsens bezüglich der Vorbereitungs‐ und Behandlungsprotokolle, der Anzahl und Intervalle der Sitzungen sowie der Dosierung erschwert.^41^ Insgesamt stellt PRP eine potenzielle Behandlungsoption für AA dar, insbesondere bei leichten Fällen.

Randomisierte kontrollierte Studien zur Wirksamkeit der PRP im Kindesalter wurden in der Literatursuche nicht identifiziert. Aufgrund der multiplen Injektionen und Schmerzhaftigkeit und keiner eindeutigen Wirksamkeitslage im Kindes‐ und Jugendalter soll die Indikation in dieser Altersgruppe nicht gestellt werden.

### Topische JAK‐Inhibitoren

**Table:** 

	Empfehlung	Empfehlungsstärke
**E52**	Topische JAK‐Inhibitoren *sollen* zur Behandlung der AA *nicht* angeboten werden.	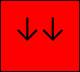
	Starker Konsens	**Evidenzbasiert**
	Referenzen: ^25^, ^29^, ^43^, ^44^, ^45^, ^46^	
	LoE: Level 1	

Die vollständigen Erläuterungen zu Wirksamkeit, Dosierung und besondere Hinweise sind in der Langversion der Leitlinie zu finden.

#### Auszug aus Wirkmechanismen und Wirksamkeit

Januskinase‐Inhibitoren (JAK‐Inhibitoren) sind eine neue Klasse von Medikamenten, die in die Signalübertragung von Zytokinen eingreifen und dadurch entzündliche Prozesse hemmen. Im Fall der AA wird angenommen, dass JAK‐Inhibitoren die durch Zytokine vermittelte Entzündung der Haarfollikel reduzieren und so das Haarwachstum fördern.^47^

Die Wirksamkeit von topischen JAK‐Inhibitoren bei AA wurde in mehreren Metaanalysen und in dem Cochrane‐Review von Mateos‐Haro et al. 2023 beschrieben. Der Cochrane Review fasst alle JAK‐ Inhibitoren in der topischen Anwendung zusammen und bewertet sie in Bezug auf das Nachwachsen der Haare von mindestens 75 % nach 12 bis 26 Wochen (RR: 5.00; 95 %‐KI 0.25–100.89).^29^ Im Vergleich zu Placebo zeigt sich ein um das fünffach verbesserte Nachwachsen, jedoch sind die Ergebnisse statistisch nicht signifikant und das sehr weite Konfidenzintervall schränkt das Vertrauen in den Effektschätzer sehr erheblich ein.^29^

#### Delgocitinib

In einer explorativen doppelblinden Studie mit 31 Patienten mit mittelschwerer bis schwerer AA (≥ 30 % Kopfhautbeteiligung) zeigte sich nach 12 Wochen Behandlung mit Delgocitinib‐Salbe (30 mg/g, zweimal täglich) im Vergleich zu einer Vehikelsalbe keine signifikante Verbesserung im Prozentsatz der Patienten, die eine 50%ige Reduktion im SALT‐Score erreichten.^48^

#### Topisches Ruxolitinib

In einer Metaanalyse zeigte sich in randomisierten kontrollierten Studien keine Überlegenheit des topischen JAK‐Inhibitors Ruxolitinib gegenüber Placebo in Bezug auf eine 50%ige Verbesserung im SALT‐Score (RR: 1,00; 95 %‐KI: 0,31–3,18). In Nicht‐RCT erreichten sieben von 22 Patienten unter topischem Ruxolitinib oder Tofacitinib (ein selektiver JAK1‐JAK3‐Inhibitor) eine mindestens 50 %ige Verbesserung im SALT‐Score (0,28; 95 %‐KI: 0,01–0,72).^43^ In einer doppelblinden klinischen Studie wurden 78 AA‐Patienten (SALT ≥ 25) randomisiert, um zweimal täglich über insgesamt 24 Wochen 1,5 %ige Ruxolitinib‐Creme (n = 39) oder Vehikel‐Creme (n = 39) auf die betroffene Kopfhautregion und bis zu 2,5 cm in die umgebenden Bereiche aufzutragen. In Woche 24 wurde kein statistisch signifikanter Unterschied zwischen den beiden Gruppen beobachtet, 12,8 % (n = 5/39) der Patienten erreichten nach Anwendung der Ruxolitinib‐Creme oder Vehikel‐Creme eine 50 %ige Verbesserung im SALT‐Score.^49^ Obwohl Pilotstudien vor der Durchführung von klinischen Studien (RCT) die Wirksamkeit von Ruxolitinib bei der Behandlung von AA gezeigt haben, zeigen die Ergebnisse der RCT keine zufriedenstellende Wirksamkeit der Ruxolitinib‐Creme.

Eine Subgruppenanalyse in einer Metaanalyse ergab, dass ein gutes oder vollständiges Haarnachwachsen bei durchschnittlich 20,8 % der Patienten unter topischer Tofacitinib‐Therapie erreicht wurde, verglichen mit 46,5 % unter oraler Therapie, wobei dieser Unterschied statistisch nicht signifikant war (p = 0,19).^45^ Bei Kindern mit AA fand eine Metaanalyse eine Eventrate für das Haarnachwachsen von 23 % (95 %‐KI 4–48 %) bei 17 Kindern unter topischem Tofacitinib 2 %.^25^ In einer weiteren Metaanalyse mit 69 Kindern sprachen 71 % der Kinder auf die topische JAK‐Inhibitor‐Therapie an, während 28 % keine Reaktion zeigten.^44^

#### Besondere Hinweise

Die Evidenz zur Wirksamkeit von topischen JAK‐Inhibitoren bei AA ist begrenzt und teilweise widersprüchlich. Die meisten Studien waren Beobachtungsstudien oder Fallserien ohne Kontrollgruppen, was die Aussagekraft der Ergebnisse einschränkt. Zudem wurden vier von acht registrierten klinischen Studien zu topischen JAK‐Inhibitoren bei AA vorzeitig aufgrund mangelnder Wirksamkeit abgebrochen.^46^

### Laser‐Verfahren

In diesem Abschnitt werden die folgenden Laser‐Verfahren beschrieben: Excimer Laser, Excimer Licht, fraktionierter CO_2_‐Laser, und die Low‐Level‐Lasertherapie (LLLT). Für diese Verfahren wurden in der Literatursuche Studien, die den Einschlusskriterien entsprachen, identifiziert. Andere Laserverfahren wurden in der Suche zur Behandlung der AA nicht identifiziert. Laserverfahren wie der Excimer Laser oder Excimer Licht, der fraktionierte CO_2_‐Laser und die *Low‐Level*‐Lasertherapie werden als nicht‐invasive Behandlungsoptionen eingesetzt.

**Table:** 

	Empfehlung	Empfehlungsstärke
**E53**	Der Einsatz der gezielten UVB‐Phototherapie (Excimer Laser oder Excimer Licht) *kann* bei Kindern, Jugendlichen und Erwachsenen mit allen Formen der AA im *Off‐Label‐Use* erwogen werden.	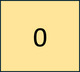
	Starker Konsens	**Evidenzbasiert**
	Referenzen: ^4^, ^25^, ^50^, ^51^, ^52^, ^53^	
	LoE: Level 1, Level 2*, Level 2	

**Table:** 

	Statement	
**S3**	Auf der Basis der identifizierten Evidenz kann die Leitliniengruppe keine Empfehlung für die Anwendung von hochenergetischen und Low‐Level‐Lasersystemen (mit Ausnahme der Excimer‐Therapie, siehe Empfehlung E 53) zur Behandlung der AA bei Kindern, Jugendlichen und Erwachsenen aussprechen.	
	Starker Konsens	**Evidenzbasiert**
	Referenzen: ^4^, ^25^, ^51^, ^52^, ^55^, ^56^	
	LoE: Level 1, Level 2*, Level 2	

Die vollständigen Erläuterungen zu Wirksamkeit, Dosierung und besondere Hinweise sind in der Langversion der Leitlinie zu finden.

#### Auszug aus Wirkmechanismen und Wirksamkeit

Zur Unterscheidung der identifizierten Technologien hier einige Hinweise: Die *Low‐Level*‐Lasertherapie (LLLT), die auch als Kaltlasertherapie bezeichnet wird, arbeitet mit geringerer Leistung und stützt sich in erster Linie auf photochemische Effekte, um zelluläre Reparaturmechanismen zu stimulieren. Die Lasertherapie der Klasse IV hingegen nutzt höhere Leistungsdichten, um eine tiefere Gewebedurchdringung zu erreichen, und kann sowohl photochemische als auch photothermische Effekte erzeugen.^57^

Die LLLT ist eine von der Food and Drug Administation (FDA) zugelassene Behandlung der androgenetischen Alopezie (AGA). Sie nutzt rotes Licht, um den Zellstoffwechsel zu stimulieren, die Haarfollikel in die Anagenphase (Wachstumsphase) zu versetzen und diese Phase zu verlängern, was das Haarwachstum fördert.^58^

Beim ablativen fraktionierten Laser‐*Resurfacing* werden mit Hilfe von Laserenergie mikrothermische Zonen geschaffen, die durch eine schnelle Wundheilung eine Hautremodellierung ohne Narbenbildung bewirken. Der Prozess umfasst Entzündungen, Zellmigration und die Bildung von Gewebe, wie zum Beispiel Neokollagen. Es werden Erbium‐dotierte Yttrium‐Aluminium‐Granat (Er:YAG)‐ und Kohlendioxid (CO_2_)‐Laser verwendet, wobei programmierbare Parameter den Koagulationsbereich, die Eindringtiefe und die Erholungszeit steuern.^58^

Excimer‐Laser, die in der Regel eine Kombination aus einem Edelgas (wie Argon oder Xenon) und einem Halogen (wie Chlor oder Fluor) verwenden, erzeugen ultraviolettes (UV) Licht mit einer Wellenlänge von 308 nm. Neben dem Excimer‐Laser gibt es mittlerweile auch nicht auf Lasertechnologie basierende Excimer‐Lichtsysteme, die ebenfalls hochintensives Licht mit einem Maximum bei 308 nm emittierten. Es wird vermutet, dass die Excimer‐Laser‐ beziehungsweise Excimer‐Licht‐Therapie eine Apoptose (programmierter Zelltod) in T‐Zellen auslöst. Indem sie die Zahl der T‐Zellen und die Antigenpräsentation verringert, kann die Excimer‐Bestrahlung dazu beitragen, immunvermittelte Hautkrankheiten wie Psoriasis und AA zu stabilisieren.^59^

Die Metaanalyse von Lee et al. 2020 mit 129 AA‐Patientinnen und Patienten basierend auf neun prospektiven Studien, vier davon ohne Vergleichsgruppe, untersuchte die Wirksamkeit und Sicherheit der Excimer‐Laser oder Excimer Lichttherapie im Vergleich zu einer Placebo‐Behandlung. Die Anwendung erfolgte in fünf Studien zweimal wöchentlich, einmal wöchentlich in zwei Studien oder alle zwei Wochen in zwei Studien. Die Behandlungsdauer lag im Median bei 12 Wochen und reichte von 4 bis 62 Wochen. Insgesamt erreichten 50,2 % (95 %‐KI 31,5 %–68,9 %) der Patientinnen und Patienten unter der Excimer‐Therapie ein Haarnachwachsen von mehr als 75 %. Bei Patientinnen und Patienten mit lokalisierter AA lag die Rate bei 56,8 % (95 %‐KI: 34,7 %–79,1 %). Die Wahrscheinlichkeit für ein Haarnachwachsen von mehr als 75 % war unter Lasertherapie 7,8‐mal höher als unter Placebo (RR: 7,83; 95 %‐KI: 2,11–29,11).^50^ In die Berechnungen der Metaanalyse wurden Daten der Excimer‐Laser‐Behandlung und der Excimer‐Licht‐Behandlung eingeschlossen ohne eine Differenzierung der beiden Methoden.^50^

Eine randomisierte klinische Studie aus dem Jahr 2022, die nicht in der oben genannten Metaanalyse enthalten ist, vergleicht die Wirksamkeit des 308‐nm‐Excimer‐Laser‐Verfahrens (XTRAC^®^; Ultra PhotoMedex) mit der Verabreichung von intraläsionalem Kortikosteroid. Die mit Excimer‐Laser behandelten Hautstellen wurden in regelmäßigen Abständen einmal pro Woche über einen Zeitraum von 12 Sitzungen (3 Monate) bestrahlt. Die anfängliche Bestrahlungsdosis betrug je nach Hauttyp 100–200 mJ. Wenn keine unerwünschten Ereignisse auftraten, wurde die Bestrahlungsdosis bei jeder Sitzung um 100 mJ erhöht. Sobald das Erythem 24 bis 48 Stunden lang anhielt, wurde die Dosis konstant gehalten. Wenn das Erythem länger als 48 Stunden anhielt oder mit Juckreiz einherging, wurde die Dosis in der nächsten Sitzung um 100 mJ reduziert. Wenn das Erythem schmerzhaft wurde oder mit Bläschen oder Blasen einherging, wurde die weitere Behandlung verschoben. Die ILC‐Injektionen (Triamcinolon 5 mg/mL) wurden alle 4 Wochen in den Sitzungen 1, 5 und 9 (3‐mal) verabreicht; im Allgemeinen wurden 0,1–0,2 mL pro Quadratzentimeter der betroffenen Haut injiziert. Die Patienten kamen einen Monat nach der Behandlung zur Nachuntersuchung in die Klinik zurück. Die Excimer‐Lasertherapie ist in der Wirksamkeit der ILC‐Therapie unterlegen. Bezogen auf den Endpunkt des Nachwachsens der Haare von mehr als 50 % erreichten 47 % der mit Laser behandelten AA‐*Patches* eine positive Antwort auf die Therapie im Vergleich zu 66 % der Patches, die mit ILC behandelt wurden (OR 0,45, p = 0,088), allerdings ist der Unterschied statistisch nicht signifikant.^53^

Rückfälle wurden teilweise in den Studien nicht evaluiert, eine Studie zum 308‐nm Excimer Laser berichtet, dass eine durchschnittliche Verbesserung von 47,5 % nach 3 Monaten erreicht wurde, aber nur 23,7 % Verbesserung nach 6 Monaten. Diese Verringerung erklärten die Autoren aufgrund von Rückfällen.^60^

Die Metaanalyse von Shen et al. 2023 schließt 14 Studien (10 RTC und 4 prospektive klinische Studien) aus den Jahren 2014 bis 2022 ein, die Studien stammen vorwiegend aus China, aber auch aus Ägypten, Belgien und der Türkei.^54^ Insgesamt werden Daten von 890 Patienten mit einer AA eingeschlossen mit einem durchschnittlichen Alter in den Studien zwischen 24 und 43 Jahren. In die Metaanalyse fließen Daten aus zwölf Studien ein. Angaben zum Schweregrad der AA werden nicht berichtet.

In der systematischen Übersichtsarbeit von Behrangi et al. 2022 werden insgesamt zehn Studien aus den Jahren 1996–2019 bei insgesamt 329 Kindern bei einem durchschnittlichen Alter von 9,3 Jahren zusammengefasst,^25^ davon sind drei retrospektive Studien, eine prospektive Studie, ein RCT, eine Open‐Label‐Studie und eine Kohortenstudie. Bei drei eingeschlossenen Studien gab es keine Zuordnung zu einem Studiendesign. Die eingeschlossenen Studien untersuchten Kinder mit einer schweren Form der AA: AT, AU, multilokaler AA, subtotaler AA und AA ophiasis oder Kinder mit einer AA mit > 30–50 % Haarverlust. Dabei kamen folgende Laserbehandlungsarten zum Einsatz: *low‐level light/laser therapy* (LLLT) sowie eine 308‐nm‐Excimerlampe mit einer minimalen Dosis von 50 mJ/cm^2^.Die Behandlungen erfolgten zweimal pro Woche für maximal 24 Sitzungen. In zwei Studien in der Übersichtsarbeit mit insgesamt 29 Kindern wird die Wirksamkeit des Excimer‐Lasers untersucht. Das komplette Nachwachsen der Haare wurde in einer der beiden Studien von 63,6 % der Kinder erreicht, in der zweiten Studie erreichten 41,5 % ein Nachwachsen der Haare (ohne genauere Definition). In beiden Studien war die Nachbeobachtungszeit 6 Monate und in beiden Studien gab es in diesem Zeitraum kein Wiederauftreten der AA. Als unerwünschte Ereignisse werden Erytheme, Hyperpigmentation, Juckreiz, mildes Peeling der Haut bei 4/11 Kindern berichtet.

#### Besondere Hinweise

Die Lasertherapie stellt eine nichtinvasive Behandlungsoption für AA dar. Hinweise zur Wirksamkeit der Laser‐ und Excimer Lichttherapie bei AA liegen zwar vor, jedoch diskutierte die Expertengruppe sehr kritisch diese therapeutische Option wegen der UV‐Strahlen‐ Exposition der Excimer‐Therapie besonders bei Kindern und Jugendlichen. Daher entschied sie sich auch bei vorliegenden Hinweisen zur Wirksamkeit für eine bedingte Empfehlung. Begründet wird diese Entscheidung darüber hinaus durch die kleinen Fallzahlen in den meisten Studien, deren kurze Nachbeobachtungszeiträume und die vorliegenden heterogenen Behandlungsprotokolle. Es sind weitere gut konzipierte randomisierte kontrollierte Studien mit größeren Patientinnen‐ und Patientenkohorten und Langzeit‐Follow‐up erforderlich, um die Ergebnisse zu bestätigen und die optimalen Behandlungsparameter zu definieren.

Die *Low‐Level*‐Lasertherapie hat sich bei verschiedenen medizinischen Anwendungen als eventuell nützlich erwiesen, unter anderem bei der Stimulierung des Haarwachstums, doch gibt es potenzielle Bedenken hinsichtlich ihrer Anwendung. Einige Studien deuten darauf hin, dass LLLT die Vermehrung bestimmter Krebszellen verstärken kann, auch wenn derzeit noch keine endgültigen Daten vorliegen.^61^, ^62^

In der Aufklärung werden daher mögliche Risiken sorgfältig erläutert, insbesondere bei Patientinnen und Patienten mit bösartigen Hauterkrankungen in der Anamnese.

### UVA1‐Phototherapie und Photochemotherapie (PUVA)

**Table:** 

	Empfehlung	Empfehlungsstärke
**E54**	Eine UVA1‐Therapie *sollte* Kindern, Jugendlichen und Erwachsenen mit allen Formen der AA *nicht* angeboten werden.	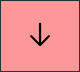
	Starker Konsens	**Evidenzbasiert**
	Referenzen: ^63^, ^64^, ^65^, ^66^	
	LoE: Level 1, Level 2*, Level 2	

**Table:** 

	Empfehlung	Empfehlungsstärke
**E55**	Eine PUVA‐Therapie *sollte* Kindern, Jugendlichen und Erwachsenen mit allen Formen der AA *nicht* angeboten werden.	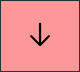
	Mehrheitliche Zustimmung	**Evidenzbasiert**
	Referenzen: ^4^, ^65^, ^66^, ^67^, ^68^	
	LoE: Level 2*, Level 2	

Die vollständigen Erläuterungen zur Wirksamkeit, Dosierung und besondere Hinweise sind in der Langversion der Leitlinie zu finden.

### Topische Therapie spezieller Lokalisationen

In der folgenden Übersicht sind die Empfehlungen zur topischen Therapie spezieller Lokalisationen zusammengefasst.

**Table:** 

		Empfehlungsstärke
**E56**	Bei AA der Augenbrauen *sollten* in diesem Bereich topische Kortikosteroide (Klasse II–III, maximal 3 Monate) oder topische Calcineurininhibitoren im *Off‐Label‐Use* angeboten werden.	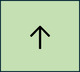
**E57**	Bei AA der Augenbrauen *können* von in der Anwendung erfahrenen Ärztinnen und Ärzten in diesem Bereich intraläsionale Kortikosteroide (2,5 mg/ml Triamcinolonacetonid 0,1 ml/cm^2^, streng intrakutan) im *Off‐Label‐Use* angeboten werden.	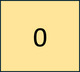
**E58**	Bei AA der Wimpern *können* in diesem Bereich im *Off‐Label‐Use* Prostaglandin‐Analoga angeboten werden.	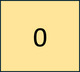
**E59**	Bei AA barbae *sollten* in diesem Bereich topische Kortikosteroide (Klasse II – III, maximal 3 Monate) oder topische Calcineurininhibitoren im *Off‐Label‐Use* angeboten werden.	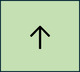
**E60**	Bei AA barbae *können* in diesem Bereich von in der Anwendung erfahrenen Ärztinnen und Ärzte intraläsionale Kortikosteroide (2,5 mg/ml Triamcinolonacetonid 0,1 ml/cm^2^, streng intrakutan) im *Off‐Label‐Use* angeboten werden.	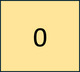
	Starker Konsens	**Konsensbasiert**

### Systemische Therapie

#### Einführung

Die verschiedenen Wirkstoffe, die in der systemischen Therapie der AA zum Einsatz kommen, sind in der Regel zur Behandlung der AA in Deutschland nicht zugelassen, können jedoch im *Off‐Label‐Use* verwendet werden. Ausnahme bilden die zwei JAK‐Inhibitoren Baricitinib und Ritlecitinib. Stand 07/2025: Für die Behandlung einer schweren Alopecia areata bei Erwachsenen (> 18. Lebensjahr) ist seit 20. Juni 2022 europaweit der JAK‐Inhibitor Baricitinib in einer Dosierung von 4 mg/Tag zugelassen.^69^

Seit 15. September 2023 hat auch der JAK‐Inhibitor Ritlecitinib in einer Dosierung von 50 mg/Tag eine EU‐weite Zulassung für die Behandlung der schweren Alopecia areata bei Jugendlichen und Erwachsenen (> 12. Lebensjahr).^70^ Beide Medikamente sind laut § 34 Abs. 1 Satz 7 SBG V verordnungsfähig, aber nicht erstattungsfähig.

Auf den *Off‐Label‐Use* wird in den Empfehlungen jeweils hingewiesen. Im Hintergrundtext der Langversion wird er aber nicht nochmals extra erwähnt.

Laut SGB V – Gesetzliche Krankenversicherung – (Artikel 1 des Gesetzes v. 20. Dezember 1988, BGBl. I S. 2477), § 34 Ausgeschlossene Arznei‐, Heil‐ und Hilfsmittel sind alle Medikamente, die „zur Verbesserung des Haarwuchses dienen“, von dieser Regelung betroffen, das heißt, dass keine Kostenübernahme durch die gesetzlichen Krankenkassen für diese Medikamente in der Therapie der AA erfolgt.^3^ Für eine Übernahme der Kosten bei Kindern, Jugendlichen und Erwachsenen kann versucht werden, individuelle Anträge auf Kostenerstattung bei den Krankenversicherungen zu stellen. Aufgrund der oben genannten Gesetzeslage wird jedoch die überwiegende Mehrheit der individuellen Anträge auf Kostenerstattung von den Krankenversicherungen abgelehnt.

Die Leitliniengruppe hat die folgenden allgemeinen Therapiegrundsätze zur systemischen Therapie verabschiedet. Sie werden hier in der Reihenfolge nach Altersgruppen gezeigt, die eine Abweichung in der Nummerierung zur Folge hat im Vergleich zur Langversion der Leitlinie.

### Allgemeine Therapiegrundsätze

**Table:** 

		Empfehlungsstärke
**E8**	*Bei Kindern* mit AA sollte eine systemische Therapie in ausgewählten Einzelfällen, nach besonderer Abwägung von Nutzen und Risiken, erfolgen.	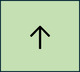
	Starker Konsens	

**Table:** 

		Empfehlungsstärke
**E7**	Die Einleitung einer systemischen Therapie sollte *bei Jugendlichen* mit AA in den folgenden Situationen angeboten werden:	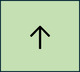
	− Ab einer moderaten AA mit hohem Leidensdruck/ausgeprägter Einschränkung der Lebensqualität bei ausbleibendem Ansprechen topischer Therapieoptionen über 6 Monate	
	− Bei schwerer bis sehr schwerer AA ab einem Bestehen länger als 6 Monate	
	Bei innerhalb von Tagen bis Wochen rasch progredienter AA	
	Starker Konsens	

**Table:** 

		Empfehlungsstärke
**E6**	Die Einleitung einer systemischen Therapie sollte *bei Erwachsenen* mit AA in den folgenden Situationen angeboten werden:	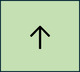
	− Patientinnen und Patienten ab einer moderaten AA bei ausbleibendem Ansprechen topischer Therapieoptionen über 6 Monate	
	− Bei schwerer bis sehr schwerer AA, die länger als 6 Monate besteht	
	− Bei innerhalb von Tagen bis Wochen rasch progredienter AA	
	− Patientinnen und Patienten ab einer moderaten AA bei hohem Leidensdruck / ausgeprägter Einschränkung der Lebensqualität	
	− Bei AA mit schwerer Nagelbeteiligung	
	Starker Konsens	

**Table:** 

		Empfehlungsstärke
**E9**	Bei *unzureichendem* Ansprechen auf eine topische oder systemische Therapie über einen Zeitraum von 6 Monaten *soll* das therapeutische Vorgehen reevaluiert und ein Wechsel auf eine alternative Therapie angeboten werden.	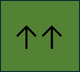
	Starker Konsens	

Tabelle 2 zeigt alle systemischen Behandlungsmöglichkeiten, getrennt nach Altersgruppen und Schweregrad der AA mit den unterschiedlichen Empfehlungsstärken. Da nur vier Behandlungsoptionen mit einem Empfehlungsgrad „kann“ oder „sollte“ ausgewiesen wurden, werden im Folgenden nur diese vier Behandlungsoptionen ausführlicher beschrieben. Alle anderen Therapien werden auf Basis der ausgewerteten Literatur und der Expertenbewertung nicht empfohlen, die ausführliche Begründung kann zu jedem Wirkstoff in der Langversion der Leitlinie nachgelesen werden.

**TABELLE 2:** Übersicht über alle Empfehlungen nach Wirkstoff – systemische Therapie.

Wirkstoff	Schweregrad SALT‐Score	Kinder	Jugendliche	Erwachsene
Puls‐Therapie mit Kortikosteroiden	Mild < 25 %	**↓**	**↓**	**↓**
	Moderat 25–49 %	**↓**	**↓**	**0**
	Schwer > 50–75 %	**0**	**0**	**↑**
	Sehr schwer 76–100 %	**0**	**0**	**↑**
Methotrexat (MTX)	Mild < 25 %	**↓**	**↓**	**↓**
	Moderat 25–49 %	**↓**	**↓**	**0** ^*^
	Schwer >50–75 %	**0**	**0**	**0** ^*^
	Sehr schwer 76–100 %	**0**	**0**	**0** ^*^
Cyclosporin A	Mild < 25 %	**↓↓**	**↓↓**	**↓↓**
	Moderat 25–49 %	**↓↓**	**↓↓**	**↓↓**
	Schwer >50–75 %	**↓↓**	**0** ^**^	**0** ^**^
	Sehr schwer 76–100 %	**↓↓**	**0** ^**^	**0** ^**^
Tumornekrosefaktor (TNF)‐α‐Inhibitoren	Mild < 25 %	**↓↓**	**↓↓**	**↓↓**
	Moderat 25–49 %	**↓↓**	**↓↓**	**↓↓**
	Schwer > 50–75 %	**↓↓**	**↓↓**	**↓↓**
	Sehr schwer 76–100 %	**↓↓**	**↓↓**	**↓↓**
Monoklonale Antikörper, zum Beispiel Dupilumab	Mild < 25 %	Keine Empfehlung	Keine Empfehlung	Keine Empfehlung
	Moderat 25–49 %	Keine Empfehlung	Keine Empfehlung	Keine Empfehlung
	Schwer > 50–75 %	Keine Empfehlung	Keine Empfehlung	Keine Empfehlung
	Sehr schwer 76–100 %	Keine Empfehlung	Keine Empfehlung	Keine Empfehlung
Azathioprin	Mild <25 %	Keine Empfehlung	Keine Empfehlung	Keine Empfehlung
	Moderat 25–49 %	Keine Empfehlung	Keine Empfehlung	Keine Empfehlung
	Schwer > 50–75 %	Keine Empfehlung	Keine Empfehlung	Keine Empfehlung
	Sehr schwer 76–100 %	Keine Empfehlung	Keine Empfehlung	Keine Empfehlung
Januskinase‐Inhibitoren (Baricitinib ≥ 18 Jahre, Ritlecitinib ≥ 12 Jahre, Stand 07/2025)	Mild < 25 %	Keine Empfehlung	Keine Empfehlung	Keine Empfehlung
	Moderat 25–49 %	Keine Empfehlung	Keine Empfehlung	Keine Empfehlung
	Schwer > 50–75 %	**0** ^***^	**0** ^***^	**↑**
	Sehr schwer 76–100 %	**0** ^***^	**0** ^***^	**↑**
Glycyrrhizin	Mild < 25 %	**↓↓**	**↓↓**	**↓↓**
	Moderat 25–49 %	**↓↓**	**↓↓**	**↓↓**
	Schwer > 50–75 %	**↓↓**	**↓↓**	**↓↓**
	Sehr schwer 76–100 %	**↓↓**	**↓↓**	**↓↓**

* Die systemische Therapie mit Methotrexat sollte Erwachsenen mit einer moderaten, schweren oder sehr schweren Form der AA als Kombinationstherapie mit systemischen Kortikosteroiden angeboten werden.

** Die systemische Therapie mit Cyclosporin A kann Jugendlichen und Erwachsenen mit einer schweren oder sehr schweren Form der AA mit rasch progredientem Verlauf für eine kurzzeitige Therapieintervention angeboten werden.

*** Die systemische Therapie mit JAK‐Inhibitoren kann bei Kindern und Jugendlichen mit schwerer und sehr schwerer AA bei entsprechender Zulassung in Hinblick auf die Altersgruppe angeboten werden.

Stand 07/2025: Für die Behandlung einer schweren Alopecia areata bei Erwachsenen (≥ 18. Lebensjahr) ist seit 20. Juni 2022 europaweit der JAK‐Inhibitor Baricitinib in einer Dosierung von 4 mg/Tag zugelassen.^69^

Seit 15. September 2023 hat auch der JAK‐Inhibitor Ritlecitinib in einer Dosierung von 50 mg/Tag eine EU‐weite Zulassung für die Behandlung einer schweren Alopecia areata bei Jugendlichen und Erwachsenen (≥ 12. Lebensjahr).^70^

Beide Medikamente sind laut § 34 Abs. 1 Satz 7 SBG V verordnungsfähig, aber nicht erstattungsfähig.^3^

### Systemische Kortikosteroide

**Table:** 

		Empfehlungsstärke
**E61**	Wenn eine systemische Kortikosteroidtherapie zur Behandlung der AA angeboten wird, *sollte* diese als gepulste und in aller Regel nicht als kontinuierliche Gabe im *Off‐Label‐Use* erfolgen.	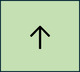
	Starker Konsens	**Evidenzbasiert**
	Referenzen: ^63^	
	LoE: Level 1	

**Table:** 

	Empfehlung	Empfehlungsstärke
**E62**	Systemische Kortikosteroide *sollten* Kindern und Jugendlichen mit einer milden oder moderaten Form der AA und Erwachsenen mit einer milden Form der AA *nicht* angeboten werden.	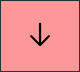
	Starker Konsens	**Evidenzbasiert**
	Referenzen: ^63^, ^71^	
	LoE: Level 1, Level 2	

**Table:** 

	Empfehlung	Empfehlungsstärke
**E63**	Systemische Kortikosteroide *können* Kindern und Jugendlichen mit einer schweren oder sehr schweren Form der AA im *Off‐Label‐Use* angeboten werden.	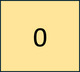
	Starker Konsens	**Evidenzbasiert**
	Referenzen: ^4^, ^71^	
	LoE: Level 2*, Level 2	

**Table:** 

	Empfehlung	Empfehlungsstärke
**E64**	Systemische Kortikosteroide *können* Erwachsenen mit einer moderaten Form der AA im *Off‐Label‐Use* angeboten werden.	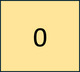
	Starker Konsens	**Evidenzbasiert**
	Referenzen: ^63^	
	LoE: Level 1	

**Table:** 

	Empfehlung	Empfehlungsstärke
**E65**	Systemische Kortikosteroide *sollten* Erwachsenen mit einer schweren oder sehr schweren Form der AA im *Off‐Label‐Use* angeboten werden.	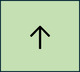
	Starker Konsens	**Evidenzbasiert**
	Referenzen: ^29^, ^63^	
	LoE: Level 1	

Die vollständigen Erläuterungen zur Wirksamkeit, Dosierung und besondere Hinweise sind in der Langversion der Leitlinie zu finden.

### Methotrexat (MTX)

**Table:** 

	Empfehlung	Empfehlungsstärke
**E66**	Die systemische Therapie mit Methotrexat *sollte* Kindern und Jugendlichen mit einer milden oder moderaten Form der AA und Erwachsenen mit einer milden Form der AA *nicht* angeboten werden.	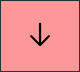
	Starker Konsens	**Evidenzbasiert**
	Referenzen: ^72^, ^73^	
	LoE: Level 2*	

**Table:** 

	Empfehlung	Empfehlungsstärke
**E67**	Die systemische Therapie mit Methotrexat *kann* Kindern und Jugendlichen mit einer schweren oder sehr schweren Form der AA im *Off‐Label‐Use* angeboten werden.	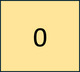
	Starker Konsens	**Evidenzbasiert**
	Referenzen: ^72^, ^73^	
	LoE: Level 2*	

**Table:** 

	Empfehlung	Empfehlungsstärke
**E68**	Die systemische Therapie mit Methotrexat *kann* Erwachsenen mit einer moderaten, schweren oder sehr schweren Form der AA als Monotherapie im *Off‐Label‐Use* angeboten werden.	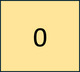
	Starker Konsens	**Evidenzbasiert**
	Referenzen: ^72^, ^73^	
	LoE: Level 2*	

**Table:** 

	Empfehlung	Empfehlungsstärke
**E69**	Die systemische Therapie mit Methotrexat *sollte* Erwachsenen mit einer moderaten, schweren oder sehr schweren Form der AA als Kombinationstherapie mit systemischen Kortikosteroiden im *Off‐Label‐Use* angeboten werden.	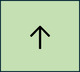
	Starker Konsens	**Evidenzbasiert**
	Referenzen: ^72^, ^73^, ^74^, ^75^	
	LoE: Level 2*, Level 2	

Die vollständigen Erläuterungen zur Wirksamkeit, Dosierung und besondere Hinweise sind in der Langversion der Leitlinie zu finden.

#### Auszug aus Wirkmechanismus und Wirksamkeit

Internationaler Konsens besteht zu folgender Empfehlung bei der Therapie mit MTX: Bei Erwachsenen sollte die Zieldosis von MTX 15 bis 25 mg pro Woche betragen. Methotrexat ist für die Behandlung der schweren und sehr schweren AA bei Jugendlichen geeignet. Wenn eine MTX‐Therapie bei Patienten unter 18 Jahren mit schwerer AA begonnen wird, sollte die Zieldosis 0,4 mg/kg/Woche betragen.^76^ Rudnicka et al. 2024 konsentierten, dass steroidsparende Mittel wie MTX nur verwendet werden sollten, um das Risiko von Nebenwirkungen einer systemischen Kortikosteroidtherapie zu verringern, wenn diese als längere Anwendung geplant sind.^77^

In der Metaanalyse von Phan et al. 2019 wurde über alle Studien hinweg ein komplettes Ansprechen auf die MTX‐Therapie von 35,8 % (95 %‐KI 25,0 %–48,3 %) errechnet. An den eingeschlossenen Studien nahmen nur Patienten mit einer schweren Form der AA teil, die entweder mindestens einen SALT‐Score von > 50 % oder eine AT oder AU hatten. Bezogen nur auf Erwachsene (n = 315) betrug der Anteil der Patienten mit einem kompletten Ansprechen auf die Therapie 44,7 % (95 %‐KI 32,9 %–57,1 %), bei Kindern (n = 58) lag dieser Anteil bei 11,6 % (95 %‐KI 5,1 %–24,5 %).^72^ Beim Vergleich der Kombination MTX plus systemischer Kortikosteroide versus *Stand‐alone*‐MTX‐Therapie zeigen die Ergebnisse der Metaanalyse (OR 2,73, 95 %‐KI 1,19–6,27),^72^ aber auch einer kleinen randomisierten kontrollierten dreiarmigen Studie (Methotrexat vs. Betamethason Pulstherapie vs. Kombination) eine Überlegenheit der Kombinationstherapie (SALT‐Score Reduktion 3 Monate nach einer sechsmonatigen Therapie um 43 %) im Vergleich zu den Monotherapien (SALT‐Score Reduktion um 26 % unter Betamethason und 23 % unter MTX).^74^

Die durchschnittliche Zeit von diffusem Nachwachsen bis zum vollständigen Nachwachsen der Haare lag in den Studien der Metaanalyse von Phan et al. 2019 zwischen 3,3 Monaten (95 %‐KI 2,3–4,0) und 9,9 Monaten (95 %‐KI 6,0–13,8). Über alle Studien hinweg mit Kindern und Erwachsenen lag die Rezidivrate (zumeist nach Dosisreduktion oder Absetzen der Therapie) für alle Patienten bei 47,7 % (95 %‐KI 35,2 %–60,5 %), bei 52,0 % (95 %‐KI 37,5 %–66,2 %) bei den Erwachsenen und bei 31,7 % (95 %‐KI 16,3 %–52,6 %) bei den Kindern.^72^

#### Kombination mit anderen Therapien

Die MTX‐Therapie in oben genannter Dosierung kann bei Erwachsenen und Kindern mit der gleichzeitigen systemischen Gabe von Steroiden^4^, ^72^ (hochdosiert zu Beginn der MTX‐Behandlung) innerhalb 1 bis 2 Wochen ausschleichend abgesetzt (nähere Angaben in Abschnitt „systemische Kortikosteroide“) beziehungsweise mit einer topischen antientzündlichen Therapie kombiniert werden.

Wechselwirkungen mit MTX mit Erhöhung der Knochenmark‐ und Lebertoxizität sind unter gleichzeitiger Einnahme mit Sulfonamiden, Barbituraten, Tetracyclinen, Retinoiden, Salicylaten, Ethanol möglich.

#### Besondere Hinweise

Methotrexat ist teratogen, daher ist eine wirksame Verhütungsmethode für weibliche Jugendliche und Frauen im gebärfähigen Alter während der Behandlung und bis zu 6 Monaten nach Abschluss der Therapie unentbehrlich. Das Gleiche gilt für Männer, die mit MTX behandelt werden. Das Stillen während der Behandlung mit MTX ist nicht zu empfehlen.

Eine Impfung mit Lebendimpfstoffen sollte während der Therapie nicht erfolgen. Weitere Impfungen sollten nur nach Rücksprache mit dem behandelnden Arzt erfolgen.

Absolute Kontraindikationen für die Behandlung mit MTX stellen eine schwere Nierenfunktionsstörung, Leberschäden, Erkrankungen des blutbildenden Systems, Alkoholabusus, Immundefizienz, schwere Infektionen, gastrointestinale Ulzera und maligne Lymphome dar.

### Cyclosporin A (Calcineurininhibitor)

**Table:** 

	Empfehlung	Empfehlungsstärke
**E70**	Die systemische Therapie mit Cyclosporin A *soll* Kindern mit allen Formen der AA und Jugendlichen und Erwachsenen mit einer milden oder moderaten Form der AA *nicht* angeboten werden.	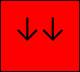
	Starker Konsens	**Evidenzbasiert**
	Referenzen: ^4^, ^28^, ^78^	
	LoE: Level 2*, Level 2	

**Table:** 

	Empfehlung	Empfehlungsstärke
**E71**	Die systemische Therapie mit Cyclosporin A *kann* Jugendlichen und Erwachsenen mit einer schweren oder sehr schweren Form der AA mit rasch progredientem Verlauf für eine kurzzeitige Therapieintervention im *Off‐Label‐Use* angeboten werden.	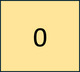
	Starker Konsens	**Evidenzbasiert**
	Referenzen: ^4^, ^28^, ^78^	
	LoE: Level 2*, Level 2	

Die vollständigen Erläuterungen zur Wirksamkeit, Dosierung und besondere Hinweise sind in der Langversion der Leitlinie zu finden.

### Monoklonale Antikörper

#### Tumornekrosefaktor (TNF)‐alpha‐Inhibitoren

**Table:** 

	Empfehlung	Empfehlungsstärke
**E72**	Die systemische Therapie mit Tumor‐Nekrose‐Faktor (TNF) α‐Inhibitoren *soll* Kindern, Jugendlichen und Erwachsenen mit allen Formen der AA *nicht* angeboten werden.	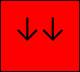
	Starker Konsens	**Evidenzbasiert**
	Referenzen: ^79^	
	LoE: Level 2*	

Die vollständigen Erläuterungen zur Wirksamkeit, Dosierung und besondere Hinweise sind in der Langversion der Leitlinie zu finden.

*Verschiedene monoklonale AK, modifiziertes Zytokin und rekombinantes Protein*

**Table:** 

	Statement	
**S4**	Auf der Basis der identifizierten Evidenz kann die Leitlinienggruppe keine Empfehlung für oder gegen die Anwendung von monoklonalen Antikörpern (außer TNF‐alpha‐Inhibitoren, siehe E82), die in Studien zur Behandlung der AA bei Kindern, Jugendlichen und Erwachsenen untersucht wurden, aussprechen.	
	Starker Konsens	**Evidenzbasiert**
	Referenzen: ^49^, ^80^, ^81^	
	LoE: Level 1, Level 2*, Level 2	

Die vollständigen Erläuterungen zur Wirksamkeit, Dosierung und besondere Hinweise sind in der Langversion der Leitlinie zu finden.

### Azathioprin

**Table:** 

	Statement	
**S5**	Auf der Basis der identifizierten Evidenz *kann* die Leitlinienggruppe *keine Empfehlung* für die Anwendung von Azathioprin zur Behandlung der AA bei Kindern, Jugendlichen und Erwachsenen aussprechen.	
	Konsens	**Evidenzbasiert**
	Referenzen: ^4^, ^29^, ^82^, ^83^	
	LoE: Level 1, Level 2*, Level 2	

Die vollständigen Erläuterungen zur Wirksamkeit, Dosierung und besondere Hinweise sind in der Langversion der Leitlinie zu finden.

### Januskinase (JAK)‐Inhibitoren

**Table:** 

	Empfehlung	Empfehlungsstärke
**E73**	Eine systemische Therapie mit JAK‐Inhibitoren *kann* bei Kindern und Jugendlichen mit schwerer und sehr schwerer AA bei entsprechender Zulassung in Hinblick auf die Altersgruppe angeboten werden.	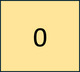
	Starker Konsens	**Evidenzbasiert**
	Referenzen: ^44^, ^84^, ^85^, ^86^	
	LoE: Level 1, Level 2*	

**Table:** 

	Empfehlung	Empfehlungsstärke
**E74**	Eine systemische Therapie mit JAK‐Inhibitoren *sollte* bei Erwachsenen mit schwerer und sehr schwerer AA angeboten werden.	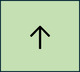
	Starker Konsens	**Evidenzbasiert**
	Referenzen: ^29^, ^43^, ^85^, ^86^, ^87^	
	LoE: Level 1, Level 2*	

Für die Behandlung einer schweren AA bei Erwachsenen (> 18. Lebensjahr) ist seit 20. Juni 2022 europaweit der JAK‐Inhibitor Baricitinib in einer Dosierung von 4 mg/Tag zugelassen.^69^

Seit 15. September 2023 hat auch der JAK‐Inhibitor Ritlecitinib in einer Dosierung von 50 mg/Tag eine EU‐weite Zulassung für die Behandlung einer schweren AA bei Jugendlichen und Erwachsenen (> 12. Lebensjahr).^70^

Beide Medikamente sind laut § 34 Abs. 1 Satz 7 SBG V verordnungsfähig, aber nicht erstattungsfähig.

Die vollständigen Erläuterungen zur Wirksamkeit, Dosierung und besondere Hinweise sind in der Langversion der Leitlinie zu finden.

#### Auszug aus Wirkmechanismen und Wirksamkeit

Inhibitoren der Januskinase wie Baricitinib, Ritlecitinib, Tofacitinib, Deuruxolitinib, Ruxolitinib, Brepocitinib sind kleine Moleküle (*small molecules*), welche die intrazelluläre Zytokintransduktion durch Blockierung des JAK‐STAT‐Signalweges (JAK‐*signal transducers and activator of transcription* [STAT]) unterbinden können und somit immunmodulierend, antiproliferativ und entzündungshemmend wirken können.^88^ Einige JAK‐Inhibitoren inhibieren selektiv (Upadacitinib und Abrocitinib [JAK 1], Brepocitinib [JAK 1/TYK 2], Ritlecitinib [JAK 3 und TEC]) und andere weniger selektiv (Tofacitinib [JAK 1,3>2], Deuruxolitinib, Ruxolitinib und Baricitinib [JAK 1, 2]), was auf deren Isotypspezifität (abhängig von ATP‐Bindungsregion) beruht. Anhand deren Bindungsmethoden und Interaktionen können die JAK‐Inhibitoren auch in reversible (kompetitive) und irreversible (kovalente) Inhibitoren (wie zum Beispiel Ritlecitinib) subklassifiziert werden.^88^

In dem Cochrane‐Review von Mateos‐Haro et al. 2023 wurde für den Endpunkt > 75 % Nachwachsen der Haare als *Short‐Term*‐Endpunkt (zwischen 12 und 26 Wochen Follow‐up) eine Risk Ratio (RR) von 7,54 (95 %‐KI 3,90–14,58) errechnet, die eine deutliche Überlegenheit im Vergleich zu einer Placebotherapie zeigte. Auch für den Endpunkt des > 75 % Nachwachsens der Haare als *Long‐Term*‐Endpunkt (> 26 Wochen Nachbeobachtung) zeigte sich diese deutliche Überlegenheit (RR 8,49 [95 %‐KI 4,70–15,34]).^29^

Die gepoolten Ergebnisse der Metaanalyse von Mao et al. 2023 (aus 5 RCT mit insgesamt 1455 Patienten aus klinischen Studien der Jahre 2016‐2022; AA‐Typen nicht immer berichtet) mit unterschiedlichen JAK‐Inhibitoren (Baricitinib, Ritlecitinib, Tofacitinib, Ruxolitinib, ATI‐501) zeigen, dass mit der Therapie der JAK‐Inhibitoren eine fünffach erhöhte Wahrscheinlichkeit für das Erreichen des SALT‐Scores 50 erzielt wurde (OR 5,08 (95 %‐KI 3,49–7,38) im Vergleich zur Placebo‐Behandlung.^85^ Für das Erreichen des SALT‐90‐Wertes lag die Wahrscheinlichkeit noch höher (OR 7,40 [95 %‐KI 4,34–12,67]).^85^

Informationen über die Wirksamkeit und Sicherheit einzelner JAK‐Inhibitoren (Baricitinib, Deuruxolitinib, Brepocitinib, Ritlecitinib, Tofacitinib) bei AA‐Erkrankten liefert die systematische Übersichtsarbeit von Sedeh et al. 2023, in welcher 37 Studien (mit Metaanalyse aus 5 RCT; Durchschnittsalter der Patienten von 4,3 bis 66 Jahre) aus den Jahren 2016 bis 2022 eingeschlossen sind,^86^ und die Metaanalyse von Wei D et al. 2023 mit vier RCT aus den Jahren 2019 bis 2022 mit insgesamt 1689 Patienten.^87^

Orales Baricitinib erwies sich als wirkungsstärker im Vergleich zu den restlichen JAK‐Inhibitoren bei Patienten mit jeglichen AA‐Formen. Baricitinib 4 mg 1 x täglich ist den anderen Behandlungsoptionen Brepocitinib und Ritlecitinib in drei Studien (n = 357) mit Endpunkt SALT_50_ (Baricitinib: *Risk Difference* [RD] 63 %, 95 %‐KI 44–82 im Vergleich zu Brepocitinib: RD 47, 95 %‐KI 32–62; und Ritlecitinib: RD 33 %, 95 %‐KI 19–47) sowie Endpunkt SALT_75_ (Baricitinib: RD 45 %, 95 %‐KI 25–65 im Vergleich zu Brepocitinib: RD 38 %, 95 %‐KI 24–53 und Ritlecitinib: RD 25 %, 95 %‐KI 12–38) überlegen.^86^

Für Patienten mit einer schweren AA (SALT‐Score > 50) zeigt sich eine Überlegenheit für orales Deuruxolitinib 12 mg 2 x täglich in zwei Studien, in denen das Erreichen des SALT_50_‐Scores (Deuruxolitinib 12 mg: RD 48 %, 95 %‐KI 30–66 im Vergleich zu Baricitinib 4 mg: RD 42 %, 95 %‐KI 35–49) und des SALT_75_ (Deuruxolitinib 12 mg: RD 34 %, 95 %‐KI 16–51 im Vergleich zu Baricitinib 4 mg: RD 31 %, 95 %‐KI 25–37) untersucht wurde.^86^

Aus der systemischen Übersichtsarbeit von Yan et al. 2022 (mit 14 Studien aus den Jahren 2016 bis 2021 mit insgesamt n = 1845 Patienten mit AA, AU, oder AT im Alter von 27,8 bis zu 47,6 Jahren) geht die deutliche Überlegenheit der JAK‐Inhibitoren nicht nur gegenüber anderen Therapieoptionen (gutes Ansprechen in RCT definiert als SALT_50_‐Werte: RR: 6,86; 95 %‐KI: 2,91–16,16 und in Beobachtungsstudien 63 % [95 %‐KI: 44 %–80 %]) hervor, sondern auch gegenüber topisch (Ruxolitinib oder Tofacitinib) oder sublingual (Tofacitinib) verabreichten JAK‐Inhibitoren.^43^

Auch bei Kindern und Jugendlichen mit AA zeigen sich JAK‐Inhibitoren wirkungsvoll und sicher. In der Metaanalyse von Chen et al. 2022 mit zehn Studien aus den Jahren 2017 bis 2022 mit insgesamt 69 Patienten im Alter von 3 bis 18 Jahren mit AA, AU oder AT und einer Erkrankungsdauer von 1 bis 15 Jahren wurde ein generelles Ansprechen (*any positive response*) auf die JAK‐Inhibitoren‐Therapie (Tofacitinib oral oder topisch und Ruxolitinib oral) von 81,9 % (95 %‐KI 69,9 %–91,9 %) berechnet.^44^ Unter Tofacitinib oral zeigte sich ein durchschnittliches Erreichen des Nachwachsens der Haare (overall hair regrowth rate) von 49 % (95 %‐KI 29 %–69 %) bei 59 Patienten im Alter von 3 bis 18 Jahren mit AA, AT und AU.^44^ Die Metaanalyse von Behrangi et al. 2022a (sieben Studien zu Tofacitinib aus dem Zeitraum von 2017 bis 2021) berichtete, dass 19 % (95 %‐KI 6 %–36 %) kaum bis kein Nachwachsen hatten und bei 12 % (95 %‐KI 1 %–29 %) es zu einer Verstärkung der AA‐Erkrankung unter der Therapie kam.^84^

#### Auszug aus Sicherheit

Die häufigsten unerwünschten Wirkungen unter JAK‐Inhibitoren (Baricitinib, Ritlecitinib, ATI‐501, Tofacitinib, Ruxolitinib) bei AA‐Erkrankten waren Infektionen, abnormale Laborwerte, neurologische Symptome, gastrointestinale Reaktionen und Hautsymptome.^85^

Sedeh et al. 2023 berichtete im Detail die Nebenwirkungen einzelner JAK‐Inhibitoren. Atemwegsinfektionen, Nasopharyngitis, Harnwegsinfektionen, Herpes‐Zoster‐Infektionen wurden unter Tofacitinib, Baricitinib und Ruxolitinib am meisten angegeben. Infektionen der oberen Atemwege und Nasopharyngitis sind häufig auch unter Deuruxolitinib, Ritlecitinib und Brepocitinib. Kopfschmerzen überwiegen unter Deuruxolitinib, Baricitinib, Ritlecitinib und Brepocitinib.^86^

Zu kutanen Ereignissen wie Akne, akneiformen Eruptionen und Follikulitis ist es unter den meisten Therapien mit JAK‐Inhibitoren (Tofacitinib, Deuruxolitinib, Baricitinib, Delgocitinib, Ruxolitinib, Ritlecitinib) gekommen (Akne 3 x häufiger unter Baricitinib gegenüber Kontrollgruppe [RR 3,48; 95 %‐KI 1,55–7,82]).^43^ Übelkeit und/oder Durchfall sind unter Deuruxolitinib, Baricitinib, Ritlecitinib und Brepocitinib berichtet. Ein Anstieg der Kreatinphosphokinase beziehungsweise Kreatinkinase im Blut ist unter Baricitinib und Deuruxolitinib angegeben, Herpes‐simplex‐Infektionen als unerwünschte Nebenwirkung besonders unter Baricitinib. Gewichtszunahme, Müdigkeit sind vorrangig unter Ruxolitinib und Konjunktivitis, Anstieg der Alanin‐Transaminase (ALT), der Aspartat‐Aminotransferase (AST), des Cholesterins und der Triglyceride (bei Kindern auch Eosinophilie [n = 5/49, 10.2 %])^44^ allein unter Tofacitinib berichtet. Blutbildveränderungen im Sinne einer Leukopenie kommen unter einer Behandlung mit Tofacitinib und Ruxolitinib vor.^86^

Die systematische Übersichtsarbeit von Papierzewska et al. 2023 berichtet, dass vereinzelt unter Tofacitinib Hyperseborrhoe (4,1 %), unter Baricitinib Hypercholesterinämie und Zellulitis und unter Brepocitinib Rhabdomyolyse aufgetreten ist.^89^ In zwei Phase‐3‐Studien mit Baricitinib vs. Placebo traten bei n = 22 Patienten schwerwiegende unerwünschte Ereignisse auf (2,4 % vs. 1,6 %, OR 1,5) im Sinn von akutem Myokardinfarkt (0,1 % vs. 0 %, OR 1,23), ventrikulärer Tachykardie (0,1 % vs. 0 %, OR 1,23), Bluthochdruck (0,1 % vs. 0 %, OR 1,23), akute Cholezystitis (0,2 % vs. 0,3 %, OR 0,8), Pyelonephritis (0,2 % vs. 0 %, OR 2,1), Knochenbrüche (0,7 % vs. 0,3 %, OR 2,5), B‐Zell‐Lymphom (0,1 % vs. 0 %, OR 1,23), Guillain‐Barré‐Syndrom (0,1 % vs. 0 %, OR 1,23), Abort (0,1 % vs. 0 %, OR 1,23). Todesfälle wurden nicht berichtet.^89^

Während des Zeitraums der Studien aus den Jahren 2016 bis 2022 in der Metaanalyse von Mao et al. 2023 traten unter der Therapie mit JAK‐Inhibitoren keine Fälle von Malignität oder Tuberkulose auf,^85^ allerdings lag der Behandlungszeitraum in den eingeschlossenen klinischen Studien nur zwischen 24 und 36 Wochen und in den Beobachtungsstudien zwischen 4 und 42 Monaten. Unter Baricitinib wurde in zwei Phase‐3‐Studien ein Fall (n = 1/22) mit B‐Zell‐Lymphom (0,1 % vs. 0 %, OR 1,23),^89^ und in dem BRAVE‐AA2‐trial ein Plattenepithelkarzinom (0,5 %, IR = 0,5) nach 16 Monaten mit Baricitinib 2 mg und ein duktales Karzinom in situ (0,4 %, IR = 0,3) nach 10 Monaten mit Baricitinib 4 mg gemeldet.^69^

Unter Ritlecitinib in der Phase‐2b/3‐Studie wurden zwei maligne Erkrankungen berichtet: Brustkrebs in der 50‐mg‐Gruppe in Verbindung mit der Behandlung nach Aussage des Prüfarztes beschrieben sowie Brustkrebs in der 200 mg + 50 mg Gruppe, der nicht in Verbindung mit der Behandlung gesehen wurde.^70^

Bei der oralen Baricitinib, Tofacitinib‐ und Ruxolitinib‐Behandlung traten signifikant geringere Raten behandlungsbedingter unerwünschter Ereignisse im Vergleich zur herkömmlichen Kortikosteroidbehandlung auf (MD 0,08, 95 %‐KI 0,02–0,42; MD 0,14, 95 %‐KI 0,04–055; MD 0,35, 95 %‐KI 0,14–0,88).^87^

Jugendliche, die keine Vorgeschichte von Windpocken haben, sollten gegen Windpocken geimpft werden, wurde von den Experten ausdrücklich angemerkt.^90^ Auch für Erwachsene vor Einleitung einer Therapie mit JAK‐Inhibitoren ist eine Herpes‐Zoster‐Impfung zu empfehlen, auch wenn eine Erstattung dieser Impfung durch die GKV erst ab dem 60. Lebensjahr erfolgt, lediglich bei Personen mit erhöhter Gefährdung kann eine Erstattung ab dem 50. Lebensjahr erfolgen. Die Herpes‐Zoster‐Impfung hat eine Zulassung ab dem 18. Lebensjahr.

#### Monitoring

Vor der Behandlung mit JAK‐Inhibitoren ist ein Screening auf chronische Infektionen laborchemisch (zum Beispiel Hepatitis B‐/C, HIV, Tuberkulose) und radiologisch (Röntgen‐Thorax) erforderlich. Der Impfstatus ist zu überprüfen. Vor sowie während der Therapie sind Laborkontrollen von Differenzialblutbild, Leber‐ und Nierenwerte sowie der Blutfettwerte (Cholesterin, LDL, HDL, Triglyzeride) in regelmäßigen Abständen gemäß der Fachinformation durchzuführen.

### Weitere steroidsparende Therapien

**Table:** 

	Empfehlung	Empfehlungsstärke
**E75**	Die systemische Therapie mit Glycyrrhizin *soll* Kindern, Jugendlichen und Erwachsenen mit allen Formen der AA *nicht* angeboten werden.	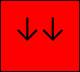
	Starker Konsens	**Evidenzbasiert**
	Referenzen: ^91^, ^92^, ^93^	
	LoE: Level 2	

Die vollständigen Erläuterungen zur Wirksamkeit, Dosierung und besondere Hinweise sind in der Langversion der Leitlinie zu finden.

### Wirkstoffe, die sich in der Behandlung der Alopecia areata als wirkungslos herausgestellt haben

*Dapson*: In der Behandlung der AA gab es vielfache Versuche, Wirkstoffe zu identifizieren, mit denen erfolgsversprechend die AA behandelt werden kann. Schon 1981 und 1995 wurde so zum Beispiel für den Wirkstoff Dapson dessen Unwirksamkeit in zwei Publikationen festgehalten.^94^, ^95^

*Etanercept/Alefacept*: Strober et al. 2005 beschreiben, dass nach einer kontinuierlichen Behandlung mit 50 mg Etanercept, das zweimal wöchentlich subkutan verabreicht wurde, über einen Zeitraum von 8 bis 24 Wochen, sich bei keinem der behandelten Probanden ein signifikantes Nachwachsen der Haare zeigte. Auf Grundlage dieser Ergebnisse, so schlussfolgern die Autoren, scheint Etanercept bei der Behandlung von Probanden mit therapieresistenter, mittelschwerer bis schwerer Alopecia areata, Alopecia totalis oder Alopecia universalis unwirksam zu sein.^96^ Strober et al. 2009 bewerteten in ihrer multizentrischen, doppelblinden, randomisierten, placebokontrollierten klinischen Studie die Wirksamkeit von Alefacept bei der Behandlung von schwerer Alopecia areata. Sie schlossen 45 Personen mit chronischer und schwerer AA, die 50 % bis 95 % der Kopfhaare betrifft und auf bisherige Therapien nicht anspricht, in ihre Studie ein. Alefacept, ein von der US‐amerikanischen Arzneimittelbehörde FDA zugelassener biologischer T‐Zell‐Hemmer zur Behandlung von mittelschwerer bis schwerer Plaque‐Psoriasis, sollte auf seine Wirksamkeit bei AA getestet werden. Als Ergebnismaß diente ein verbesserter SALT‐Score über 24 Wochen. Die Teilnehmer, die 12 Wochen lang Alefacept erhielten, zeigten im Vergleich zur Placebo‐Gruppe keine statistisch signifikante Verbesserung der AA (p = 0,70). Die Autoren schlussfolgerten, dass Alefacept zur Behandlung von schwerer AA unwirksam ist.^97^

Die aktuelle britische Leitlinie zur AA von Harries et al. 2025 hält mit den Empfehlungen dieser Leitlinie übereinstimmende Aussagen zu weiteren Wirkstoffen fest, die einmal oder wiederholt negative Ergebnisse in der Therapie der AA zeigten:

„Es gibt *keine* ausreichenden Belege, um Menschen mit AA die folgenden *systemischen Interventionen* zu empfehlen“: Inosiplex (Isoprinosin oder Inosin Pranobex), Imipramin, Apremilast, Sulfasalazin, Mesalazin, Hydroxychloroquin, Dimethylfumarat.^98^

Auch zu den Verfahren der nichtsteroidalen injizierbaren Therapien liegen laut der britischen Leitlinie keine ausreichenden Belege vor:^98^ plättchenreiches Plasma (PRP), Microneedling, Carboxytherapie, Kryotherapie, intraläsionale Pentoxifyllin‐Injektion, intraläsionale Methotrexat‐Injektion, intraläsionale Vitamin‐D‐Injektion, mesenchymale Stammzellen, intraläsionale Interferon‐alpha‐Injektion, intradermale Minoxidil‐Injektion.

Darüber hinaus hält die britische Leitlinie fest, dass es keine ausreichenden Belege gibt, um Menschen mit AA die folgenden alternativen Interventionen zu empfehlen: Aromatherapie, Allium‐/Zwiebelsalbe, Ginseng, Pfingstrose, Glycyrrhizin, kombinierte komplementäre Therapien (Kräuter, Brennnessel, Löwenzahn), giftige Primel (*Primula obconica*), Kräutersensibilisatoren, Candida‐Antigen, Squill‐Lotion und Hypnose.^98^

### Supportive Gabe von Nahrungsergänzungsmitteln

**Table:** 

	Statement	
**S6**	Auf der Basis der identifizierten Evidenz *kann* die Leitlinienggruppe *keine* Empfehlung für die Einnahme von Nahrungsergänzungsmitteln zur Behandlung der AA bei Kindern, Jugendlichen und Erwachsenen aussprechen.	
	Starker Konsens	**Evidenzbasiert**
	Referenzen: ^99^, ^100^	
	LoE: Level 2*	

Die vollständigen Erläuterungen zur Wirksamkeit, Dosierung und besondere Hinweise sind in der Langversion der Leitlinie zu finden.

### Psychologische Unterstützung

**Table:** 

	Empfehlung	Empfehlungsstärke
**E76**	Alle Patientinnen und Patienten mit allen Formen der AA, unabhängig vom Alter, *sollen* über die Möglichkeiten psychologischer Unterstützung informiert werden.	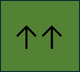
	Starker Konsens	**Konsensbasiert**

**Table:** 

	Empfehlung	Empfehlungsstärke
**E77**	Alle Patientinnen und Patienten mit allen Formen der AA, unabhängig vom Alter, *sollen* über die Angebote von Selbsthilfegruppen informiert werden.	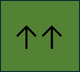
	Starker Konsens	**Konsensbasiert**

**Table:** 

	Empfehlung	Empfehlungsstärke
**E78**	Bei positiver Anamnese hinsichtlich Depression oder sozialen Ängsten *sollte* eine psychotherapeutische Diagnostik und/oder unterstützende Psychotherapie empfohlen werden.	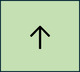
	Starker Konsens	**Konsensbasiert**

Weitere Details siehe Langversion.

### Lebensqualität im Verlauf der Therapie

Studien zur Lebensqualität von AA‐Patienten zeigen übereinstimmend eine Einschränkung, die sich unter Therapie jedoch verbessert. So gleichen sich zum Beispiel die Lebensqualitätsergebnisse von erfolgreich behandelten Patienten mit einer AA in Australien den Werten der australischen Bevölkerung an. Bei Erwachsenen zeigt sich ein deutlicher Zusammenhang mit dem Therapierfolg, je stärker die Haare nachwachsen, desto mehr verbessert sich die Lebensqualität.^75^, ^101^, ^102^, ^103^ Am deutlichsten verbesserte sich die Lebensqualität für die Patienten, die einen SALT‐Score < 20 in der Therapie erreichten.^104^

Auch Therapien, die zwar nicht das Nachwachsen der Haare beeinflussen, wie Hypnotherapie oder *mindfulness‐based stress reduction* verbessern die Lebensqualität.^105^

Für Kinder bedeutet die AA‐Erkrankung ebenfalls eine Einschränkung in ihrer Lebensqualität, die im Vergleich zu gesunden Gleichaltrigen sogar deutlich höher ist. Die systematische Übersichtsarbeit von Prendke et al. 2023 berichtet über eine geringe bis moderate Beeinträchtigung der Lebensqualität von Kindern und Jugendlichen durch die AA. Die gemessenen Mittelwerte des *Childrens Dermatology Life Quality Index* (cDLQI) lagen bei 2,2 (SD 0,96) oder 4,4 (SD 4.9) mit einem Bereich von 0–22 oder bei 6,3 (SD 5,9) in den eingeschlossenen Studien. Ein deutlicher Zusammenhang zwischen Therapieerfolg und Lebensqualität zeigte sich bei Kindern nicht, mit Beginn der Pubertät verschlechtert sich jedoch oftmals die Lebensqualität der Jugendlichen mit einer AA.^106^

### Kosmetische Angebote

**Table:** 

	Empfehlung	Empfehlungsstärke
**E79**	Alle Patientinnen und Patienten mit allen Formen der AA, unabhängig vom Alter, *sollen* über die Möglichkeiten eines Haarersatzes und über kosmetische Angebote informiert werden.	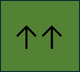
	Starker Konsens	**Konsensbasiert**

Die Expertengruppe spricht sich für eine allgemeine Information zu kosmetischen Angeboten und zu Haarersatz für alle Patientinnen und Patienten aus, um das Spektrum für Management und Krankheitsbewältigung von Anfang an in all seiner Vielfalt aufzuzeigen. Dies soll der Krankheitsbewältigung dienen und frühzeitig Ängste und Sorgen für mögliche Entwicklungen im Verlauf der AA nehmen oder vermindern.

Weitere Details, speziell Hinweise auf welche Möglichkeiten die Behandelnden aufmerksam machen können, sind in tabellarischer Form der Langversion zu entnehmen.

## DANKSAGUNG

Open access Veröffentlichung ermöglicht und organisiert durch Projekt DEAL.

## INTERESSENKONFLIKT

Eine vollständige Liste der erklärten Interessenkonflikte ist im Leitlinienreport unter https://register.awmf.org/de/leitlinien/detail/013‐104 zu finden.
